# Acute Cortical Blindness Following Acute Carbon Monoxide Intoxication Due to Gas Geyser Syndrome

**DOI:** 10.7759/cureus.70408

**Published:** 2024-09-28

**Authors:** Nirmal Pandey, Saket Nigam

**Affiliations:** 1 Neurology, Regency Hospital, Kanpur, IND; 2 Radiology, Regency Hospital, Kanpur, IND

**Keywords:** carbon monoxide (co) poisoning, diffusion-weighted imaging (dwi), liquified petroleum gas, mri- magnetic resonance imaging, neurotoxicity, occipital lobe, toxic leukoencephalopathy, visual cortex

## Abstract

Accidental carbon monoxide (CO) poisoning is common during the winters. The use of gas geysers by Indian households during this season for heating purposes may inadvertently lead to brain injury. Such cases can be misdiagnosed as seizures, resulting in inappropriate treatment. Typically, CO poisoning results in bilateral damage to the globus pallidus, while injury to the cortical gray matter is less common. A magnetic resonance imaging (MRI) of the brain done within the first 24 hours might not detect all the damage, so it's important to have a follow-up MRI within 3-7 days for a more accurate assessment. Our case presents a unique instance of CO poisoning where the patient exhibited near-symmetric posterior gray matter damage, without involvement of the basal ganglia, resulting in acute cortical blindness following exposure to a gas geyser. The awareness of this unusual and peculiar clinical and radiological presentation in gas geyser-related cases should encourage clinicians to adopt a more proactive approach.

## Introduction

During the winter months in North India, many people use instant gas geysers, an economical choice, to heat water quickly for bathing. Liquefied petroleum gas, which is used as fuel in these geysers, consumes oxygen from the atmosphere and produces carbon monoxide (CO), a colorless, odorless, and non-irritating gas that can accumulate in confined areas with poor ventilation [1]. If gas geysers are used in small, poorly ventilated bathrooms where individuals may spend extended periods bathing, or performing other tasks, such as washing clothes, while the geyser is on, it may lead to CO accumulation in these closed spaces. CO has a strong affinity for hemoglobin, more than 200 times that of oxygen. The displacement of oxygen from hemoglobin reduces the blood’s oxygen-carrying capacity, leading to progressive hypoxia in the organs it supplies. The heart and brain are particularly susceptible to hypoxia due to their high metabolic demands [2-4]. Magnetic resonance imaging (MRI) of the brain in acute CO poisoning typically reveals bilateral damage to the globus pallidus and, occasionally, the cerebral white matter [3,4]. We present an unusual case of acute CO poisoning where both the globus pallidus and cerebral white matter were notably spared.

## Case presentation

A 15-year-old previously healthy female was brought to our institution during the winter season in North India, having been found unconscious in a poorly ventilated bathroom in a nude state. She had been using a gas geyser during a hot bath, in a small poorly ventilated bathroom, and was discovered in this condition 45 minutes after entering it. Initially, she was taken to a local hospital where she received oxygen and intravenous fluids, which led to a partial recovery of consciousness. However, she remained extremely irritable and persistently cried out that she could not see. She was admitted to our facility six hours after being removed from the bathroom. Upon admission, the patient exhibited extreme agitation and restlessness, moving all four extremities symmetrically, and consistently complaining of complete blindness. Her pupils were reacting equally to light. Finger counting and light perception were not possible due to her agitation. Deep tendon reflexes were normal, with bilateral flexor plantar responses. A detailed neurological examination was not feasible due to her agitated state, which required sedation.

**Figure 1:**
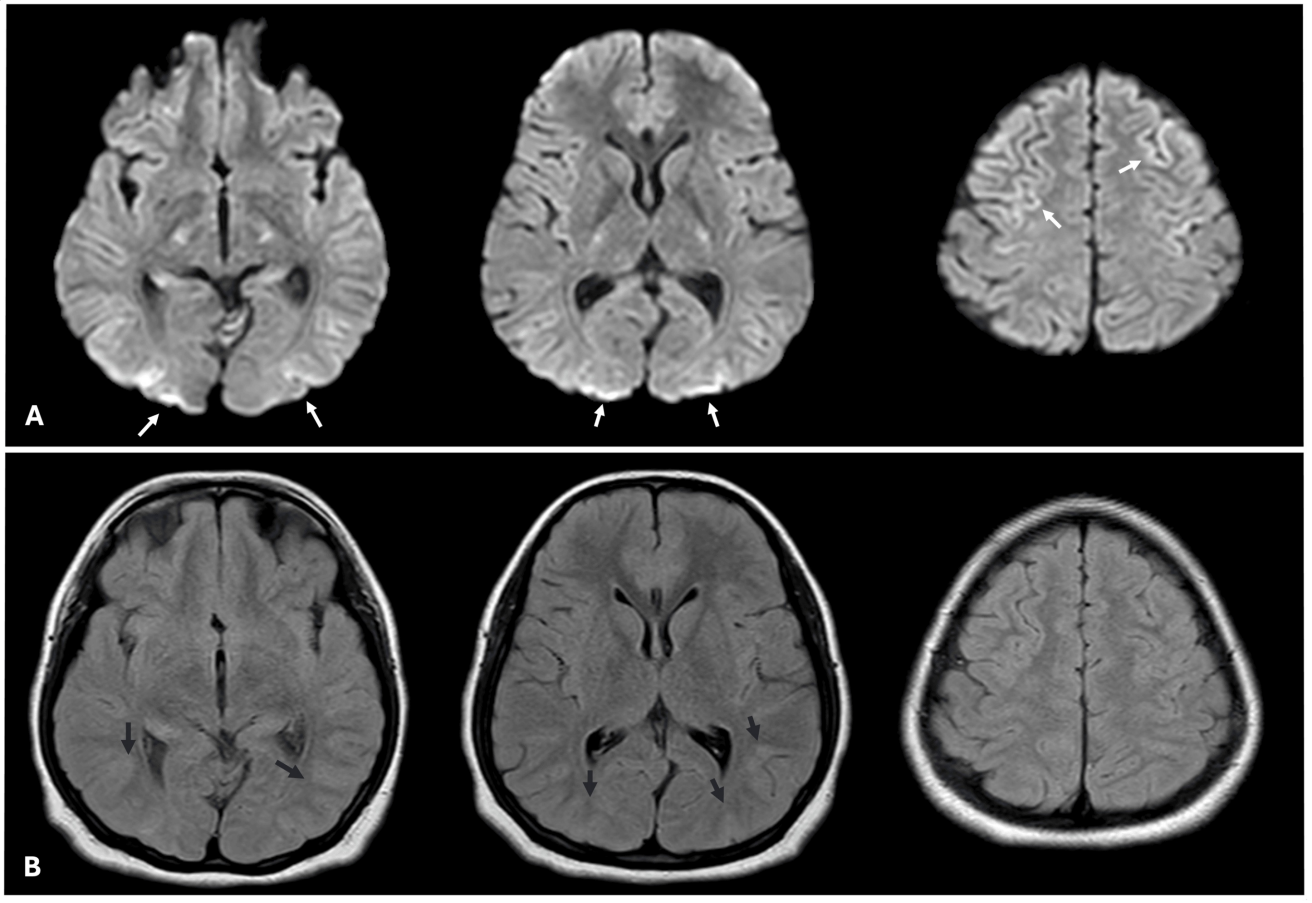
MRI findings on day 1. Faint bilateral occipital and high parietal hyperintensities (white arrows) are seen on axial DWI (A) images. Corresponding FLAIR (B) images show bilateral occipital gyral swelling (black arrows). DWI: diffusion-weighted imaging, FLAIR: fluid-attenuated inversion recovery, MRI: magnetic resonance imaging.

Initial MRI, performed within 24 hours (Day 1), revealed faint bilateral occipital gray matter and high parietal diffusion-weighted imaging (DWI) hyperintensities (Figure 1A), without apparent diffusion coefficient (ADC) restriction, accompanied by faint bilateral occipital gyral swelling on fluid-attenuated inversion recovery (FLAIR) (Figure 1B) and T2-weighted sequences. No contrast enhancement was observed. Her neurological status remained unchanged over the following three days. She continued to be highly irritable and agitated, necessitating the use of restraints and intermittent sedation as needed. Whenever she emerged from sedation, she consistently complained of complete vision loss. Throughout her hospital stay, no cardiac abnormalities were noted, and routine blood tests, including blood sugar levels, were within normal ranges.

**Figure 2:**
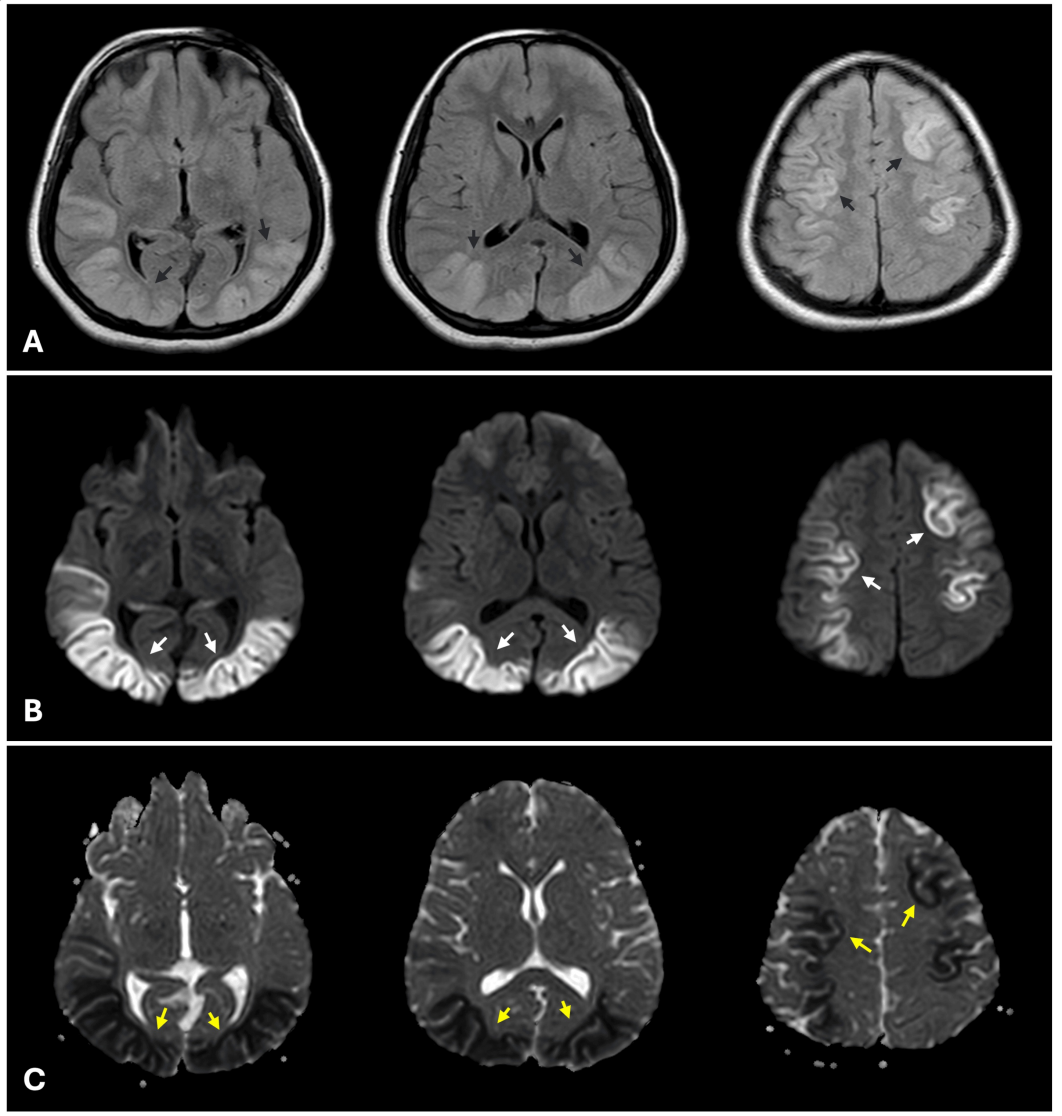
MRI findings on day 4. Axial FLAIR images (A) show extensive near-symmetrical bilateral occipital, with asymmetrical bilateral high parietal cortical gray matter hyperintensities (black arrows). Corresponding DWI (B) and ADC (C) sequences show restricted diffusion of the same regions (white and yellow arrows). ADC: apparent diffusion coefficient, DWI: diffusion-weighted imaging, FLAIR: fluid-attenuated inversion recovery, MRI: magnetic resonance imaging.

A repeat non-contrast MRI of the brain was performed 72 hours later (Day 4), revealing extensive, near-symmetrical bilateral occipital and asymmetrical high parietal cortical gray matter hyperintensities on FLAIR (Figure 2A) and T2-weighted images. These areas exhibited restricted diffusion on DWI/ADC sequences (Figure 2B, C). Brain vascular imaging was not performed during the patient's stay. The patient's relatives opted for further treatment elsewhere, precluding follow-up evaluation.

## Discussion

CO poisoning is one of the most common causes of poisoning, whether accidental or intentional. Accidental exposures particularly occur during harsh winters when gaseous heating methods are used in poorly ventilated rooms [2]. In North India, gas geyser-induced acute CO intoxication is a recognized medical emergency during the winter season. These cases are frequently mistaken as seizure episodes, leading to the inappropriate administration of anti-seizure medications. A key diagnostic clue is a history of unconsciousness while taking a hot water bath with a gas geyser switched on during winter. Many patients recover at home if rescued early, while those who reach the emergency department usually recover with oxygen supplementation. However, severe and prolonged CO exposure can, in rare cases, result in death or significant neurological disabilities. MRI of the brain is highly sensitive in detecting the extent of CO-induced brain damage. The globus pallidus is particularly vulnerable to acute CO poisoning, likely due to its poor anastomotic blood supply, or the high heme iron content, to which CO binds directly. This damage is typically observed on MRI as symmetrical lesions with restricted diffusion on DWI and hyperintensities on T2-weighted and FLAIR sequences. CO-induced brain injury may also manifest on MRI as similar bilateral lesions involving the cerebral white matter, hippocampi, caudate nuclei, putamina, thalami, cerebellum, corpus callosum, and cerebral cortex [3,4].

Cortical blindness refers to the inability to see due to bilateral damage to the occipital visual cortex. Acute cortical blindness as a presenting feature of CO poisoning caused by gas geyser use has not been well documented. In one report, similar vision loss occurred as a delayed manifestation a week after acute CO exposure, accompanied by amnesia [5]. This patient also had bilateral occipital lobe findings on MRI, along with the involvement of the hippocampi and cerebellum. In another case, the patient reported vision loss a few days after the incident, with MRI showing bilateral occipital lobe hyperintensities on DWI [6]. Posterior reversible encephalopathy syndrome (PRES), often associated with hypertension, is another frequent cause of acute altered sensorium with reversible cortical blindness, typically accompanied by seizures. MRI of the PRES usually reveals asymmetric white matter hyperintensities in the occipital and parietal lobes on FLAIR and T2-weighted images, without significant signal changes on DWI [7]. Our patient had exclusive near-symmetric gray matter involvement showing diffusion restriction. Bilateral acute posterior cerebral artery (PCA) infarction is also a common cause of acute cortical blindness. In hyperacute settings (less than 24 hours), MRI typically shows diffusion restriction in the vascular territory of the bilateral PCA, which was not observed in our case on initial imaging [7].

The isolated acute involvement of the bilateral occipital cerebral cortex, sparing the globus pallidi, cerebral white matter, and other commonly affected areas in acute CO poisoning, is a clinical enigma that has rarely been observed. The globus pallidus is more vulnerable to CO poisoning due to its poor blood supply and higher iron content, but cortical gray matter lacks these properties. While it could be theorized that the high metabolic activity and blood demand of neurons in the gray matter make them more susceptible to CO poisoning, this same high activity should theoretically make them more resistant to hypoxia over prolonged periods [3,8,9]. Like globus pallidus’ poor anastomotic blood supply, our patient may have had a preexisting precarious bilateral occipital lobe blood supply, which may have contributed to the MRI findings. However, cerebral angiography was not performed. This unique pattern of near-symmetric extensive damage to the bilateral occipital cortical gray matter, along with milder asymmetric damage to the bilateral superior parietal cortices, without frontotemporal involvement, has not been commonly reported in acute CO poisoning. A hyperacute MRI (within 24 hours), as in our case, may not fully capture the extent of hypoxic damage. Therefore, a repeat MRI after three to four days is recommended to better appreciate the areas and extent of brain injury in cases of acute CO poisoning.

## Conclusions

Acute cortical blindness caused by gas geyser-induced CO poisoning, characterized by isolated bilateral occipital cortical damage without globus pallidus involvement, is an unusual and previously undocumented phenomenon. The underlying mechanism behind this selective damage remains unclear. Clinicians should be especially vigilant about this clinico-radiographical presentation, particularly in India, where the use of gas geysers in poorly ventilated bathrooms for instant water heating during winter is commonplace. Policymakers should enforce regulations to ensure that gas geysers are not installed inside bathrooms under any circumstances.
